# Effects of Green Tea–Intake Timing on Glucose and Lipid Metabolism in Older Adults: An 8‐Week Randomized Controlled Trial

**DOI:** 10.1155/jnme/2301278

**Published:** 2026-04-07

**Authors:** Saeka Fuke, Kyoko Fujihira, Masaki Takahashi

**Affiliations:** ^1^ Department of Social and Human Sciences, Institute of Science Tokyo, Tokyo, Japan, tmd.ac.jp; ^2^ Department of Liberal Arts, Tokyo University of Technology, Tokyo, Japan, teu.ac.jp; ^3^ Institute for Liberal Arts, Institute of Science Tokyo, Tokyo, Japan, tmd.ac.jp

**Keywords:** aging, catechin, chrononutrition, glucose metabolism, green tea

## Abstract

Catechins in green tea have been reported to enhance glucose tolerance and lipid metabolism. However, the influence of chronic intake timing on these outcomes in older adults has not been fully elucidated. In this randomized controlled trial, we investigated the effects of green tea intake at different timings on glucose and lipid metabolism in older adults. Forty‐five participants aged ≥ 65 years were randomly assigned to morning (*n* = 15), day (*n* = 15), or evening (*n* = 15) group. Participants consumed green tea daily for 8 weeks at specified times (0600–1100, 1100–1600, and 1600–2100 h for the morning, day, and evening groups, respectively). Glucose metabolism, lipid metabolism, and body composition were evaluated before and after the intervention. Blood glucose level, glycated hemoglobin level, body weight, and fat mass decreased with green tea intervention, while muscle mass increased across all groups (all *p* < 0.05). These findings indicate that individuals can expect similar improvements in glucose tolerance and body composition parameters from continuous green tea consumption regardless of intake timing in older adults.

**Trial Registration:** University Hospital Medical Information Network (UMIN): UMIN000058708

## 1. Introduction

The global burden of noncommunicable diseases (NCDs) exceeded 43 million deaths in 2021, making them a major public health concern [1]. The risk of NCDs such as diabetes and cardiovascular disease increases with aging due to a progressive decline in metabolic function [2, 3]. The global population aged ≥ 60 years is projected to reach 2.1 billion worldwide by 2050, underscoring the importance of NCD‐prevention strategies in older adults [4]. The World Health Organization identifies elevated blood glucose level and abnormal lipid profiles as key risk factors for NCDs [5]. Nutritional interventions have been shown to alleviate these risks [6, 7], highlighting the need for effective dietary approaches in older adults.

Metabolic processes in mammals, including humans, follow circadian rhythms. For example, the expression of glucose transporters such as SGLT1, GLUT2, and GLUT5 peaks in the evening, while insulin secretion declines at night, leading to low glucose tolerance compared with that in the morning [8–11]. Similarly, a decrease in the expression of genes regulating fatty acid oxidation and an increase in that of lipogenesis‐related genes in the evening have been reported [12]. Meal timing also influences glucose tolerance and lipid metabolism [13]. Participants consuming high‐energy meals in the evening for 12 weeks exhibited impaired glucose tolerance compared with those consuming the same meals at breakfast despite equal total daily calorie intake [14]. Moreover, in a previous study, acute intake of 1‐deoxynojirimycin from mulberry leaf extract reduced postprandial blood glucose level more effectively at dinner than at breakfast [15]. In another study, the morning consumption of fish oil–enriched sausages for 8 weeks significantly reduced triglyceride (TG) levels [16]. These findings indicate that meal timing, governed by circadian regulation of absorption and metabolism, critically shapes dietary intervention outcomes.

Green tea, a widely consumed beverage, contains catechins with reported health benefits, including the inhibition of gluconeogenesis and enhancement of lipid metabolism [17, 18]. Interventional studies have demonstrated that green tea intake reduces fasting and postprandial blood glucose level, glycated hemoglobin (HbA1c) level, body weight (BW), and low‐density lipoprotein cholesterol (LDL‐C) level [19–21]. Interestingly, Takahashi et al. reported that acute evening consumption of green tea suppressed postprandial blood glucose level more than morning intake in healthy young male individuals [22]. This finding suggests that the metabolic effects of green tea may depend on intake timing. However, it remains unclear whether the time‐dependent effects observed in young adults by Takahashi et al. also apply to older adults. Furthermore, the long‐term effects of green tea intake timing on glucose and lipid metabolism have not been fully elucidated. Given the elevated risk of NCDs in older adults, identifying optimal intake timing may inform effective preventive strategies.

The aim of this study was to examine the effects of morning, daytime, and evening green tea consumption over 8 weeks on glucose and lipid metabolism in older adults. We hypothesized that green tea would improve the levels of glucose and lipid biomarkers regardless of intake timing but that diurnal variation in glucose tolerance and lipid metabolism might influence the magnitude of these effects.

## 2. Participants and Methods

### 2.1. Participants

**FIGURE 1:**
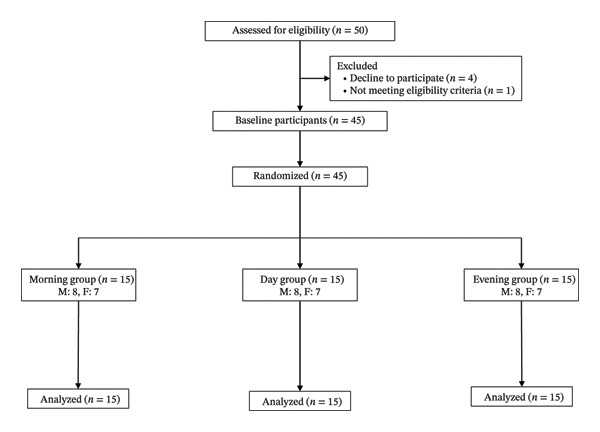
Flow diagram.

Forty‐five older adults (24 male and 21 female individuals) aged ≥ 65 years participated in this study. The exclusion criteria were a diagnosis of diabetes, renal disorder, or dyslipidemia requiring dietary restrictions; treatment for these conditions; use of antipsychotics or hormonal agents that may influence appetite; and physical complications associated with blood tests. Initially, 50 older adults (27 male and 23 female individuals) were included; however, five were excluded: one did not meet the eligibility criteria and four declined participation before baseline assessment. Finally, 45 participants were included in the final analysis (Figure 1). The study was approved by the Ethics Committee of the Institute of Science Tokyo (approval number: 2024214) and conducted in accordance with the tenets of the Declaration of Helsinki. All participants provided written informed consent. The physical characteristics of the participants are presented in Table 1.

**TABLE 1:** Participant characteristics.

			**p** **value**

Age (years)	MG	71.5 ± 3.3	0.354
	DG	70.0 ± 3.8	
	EG	70.5 ± 3.8	

Height (cm)	MG	162.4 ± 6.8	0.779
	DG	164.1 ± 9.6	
	EG	163.9 ± 8.0	

Systolic blood pressure (mmHg)	MG	151.2 ± 4.4	0.223
	DG	139.3 ± 5.1	
	EG	143.3 ± 4.5	

Diastolic blood pressure (mmHg)	MG	88.3 ± 2.8	0.834
	DG	86.2 ± 2.0	
	EG	86.5 ± 2.7	

Green tea consumption (mL/day)	MG	376.1 ± 70.7	0.836
	DG	566.7 ± 187.7	
	EG	876.7 ± 449.8	

*Note:* Values are expressed as the mean ± standard error.

### 2.2. Experimental Protocol

This randomized controlled trial was conducted in Tokyo, Japan, between March and November 2025. The 45 older adults were randomly assigned to one of the following three groups: morning group (MG), day group (DG), and evening group (EG). Randomization was performed using computer‐generated numbers in Excel by an independent researcher not involved in the trial. The intervention groups (MG, DG, and EG) consumed the test beverage daily for 8 weeks at designated times (0600–1100 h for the MG, 1100–1600 h for the DG, and 1600–2100 h for the EG) and refrained from consuming green tea outside these designated times during the study period. Participants were instructed to maintain their usual diets, except for the intake of the test beverage, and to record the date, time, and content of intake. Before and after the intervention, the participants visited the laboratory at 0830 h following a minimum 10‐h overnight fast (water permitted). After 10–15 min of rest, fasting venous blood samples were collected by venipuncture with participants seated.

### 2.3. Test Beverages

The test beverage was KOI‐MIDORI, a powdered tea manufactured by ITO EN, Ltd. (Tokyo, Japan), containing 394 mg of catechins with a galloyl moiety per two sticks (5 g). The participants were instructed to mix two sticks with 600 mL of water and consume the beverage only at assigned times. They recorded the date and time of each intake. This beverage was selected because previous studies have demonstrated significant reductions in total cholesterol, LDL‐C, and glucose‐related marker levels after 8 weeks of consuming 394.8–400 mg of tea catechins [23, 24].

### 2.4. Standardization of the Meal and Physical Activity

The participants were instructed to maintain their habitual lifestyle throughout the study. They were required to abstain from breakfast before baseline and 8‐week assessments to minimize dietary influence on body composition and blood measurements. To evaluate whether pre‐ and postintervention energy and macronutrient intake affected glucose and lipid metabolism, dietary intake was assessed using a computerized Food Frequency Questionnaire (FFQ) based on food groups (Kenpakusha, Tokyo, Japan) [25].

### 2.5. Anthropometry

Anthropometric parameters were assessed at baseline and after 8 weeks. BW, fat mass, and muscle mass were measured using a digital scale (InBody 230; InBody Japan Inc., Tokyo, Japan). Body Mass Index (BMI) was calculated as weight (kg) divided by height squared (m^2^). Arterial blood pressure was measured with a brachial electronic monitor (HCR‐7202; OMRON DALIAN Co., Ltd., Kyoto, Japan) while participants were seated. The participants rested for 5 min in a chair prior to measurement.

### 2.6. Blood Collection and Analysis

For plasma glucose and HbA1c measurements, venous blood was collected in tubes containing sodium fluoride–ethylenediaminetetraacetic acid, centrifuged at 3000 rpm for 10 min, and stored at −80°C until analysis. For serum insulin, high‐density lipoprotein cholesterol (HDL‐C), LDL‐C, and TG quantification, blood was collected in tubes containing dipotassium salt–ethylenediaminetetraacetic acid, allowed to stand at room temperature for approximately 30 min, centrifuged, and stored at −80°C. The analyses were conducted by Kotobiken Medical Laboratories (Tokyo, Japan).

### 2.7. Statistical Analysis

The analyses included participants who consumed green tea for ≥ 90% of the 56‐day intervention period. Data are expressed as the mean ± standard error. The Shapiro–Wilk test was used to assess normality of blood parameters, anthropometrics, and nutritional intake. Sample size was based on previous studies evaluating 8‐week green tea supplementation effects on BW, lipid levels, and lipid peroxidation [19]. Power calculations using G∗Power Version 3.1.9.7 indicated that 36 participants were required to achieve approximately 80% power to detect large effects at α = 0.05 [26]. Baseline group comparisons were performed using one‐way analysis of variance (ANOVA) or the Kruskal–Wallis test. Two‐way ANOVA was applied to evaluate changes in blood parameters, anthropometrics, and nutritional intake across groups before and after intervention. Statistical significance was defined as *p* < 0.05. All analyses were conducted using Predictive Analysis Software Version 23.0 for Windows (SPSS Japan Inc., Tokyo, Japan).

## 3. Results

### 3.1. Participant Characteristics

Participant characteristics are summarized in Table 1. No significant differences in age, height, blood pressure, or green tea consumption were observed among the groups at baseline.

### 3.2. Energy and Nutrient Intake

Table 2 presents changes in energy and nutrient intake after the intervention. No significant baseline differences were observed among groups. The two‐way ANOVA revealed no significant effects of time or group × time interaction on energy, protein, fat, or carbohydrate intake.

**TABLE 2:** Changes in energy and nutrient intake at baseline and after 8 weeks.

		**Baseline**	**8 weeks**	**Time**	**Time** **×** **group**

Energy (kcal)	MG	2053.7 ± 113.0	2038.8 ± 122.0	0.818	0.958
	DG	2035.7 ± 85.5	2043.6 ± 103.0		
	EG	2153.3 ± 117.6	2124.4 ± 104.6		

Protein (g)	MG	78.8 ± 6.4	79.1 ± 5.1	0.987	0.970
	DG	73.1 ± 4.2	73.6 ± 4.1		
	EG	76.2 ± 5.1	75.5 ± 4.0		

Fat (g)	MG	72.0 ± 5.4	74.3 ± 4.9	0.520	0.885
	DG	73.5 ± 4.5	73.4 ± 5.0		
	EG	77.1 ± 4.6	78.9 ± 4.6		

Carbohydrate (g)	MG	256.7 ± 14.4	250.8 ± 15.8	0.486	0.878
	DG	250.7 ± 9.5	250.6 ± 13.0		
	EG	263.5 ± 16.0	254.8 ± 14.4		

*Note:* Values are expressed as the mean ± standard error.

### 3.3. Effects of Green Tea–Intake Timing on Metabolic Parameters

Figure 2 shows changes in the glucose level, HbA1c level, insulin level, HOMA‐IR, HDL‐C level, LDL‐C level, LDL/HDL ratio, and TG level after the intervention. The two‐way ANOVA results indicated no significant effects of time or group × time interaction for glucose level, HbA1c level, insulin level, HOMA‐IR, LDL‐C level, HDL‐C level, LDL/HDL ratio, or TG level. However, significant main effects of time were detected for glucose and HbA1c levels.

FIGURE 2Changes in (a) glucose level, (b) HbA1c level, (c) insulin level, (d) HOMA‐IR, (e) HDL‐C level, (f) LDL‐C level, (g) LDL/HDL ratio, and (h) TG level after the intervention. Values are presented as the mean ± standard error at baseline and 8 weeks for each group. MG, morning group; DG, day group; EG, evening group; HbA1c, glycated hemoglobin; HOMA‐IR, homeostasis model assessment of insulin resistance; HDL‐C, high‐density lipoprotein cholesterol; LDL‐C, low‐density lipoprotein cholesterol; LDL/HDL ratio, LDL‐C/HDL‐C ratio; TG, triglyceride.

**Figure:**
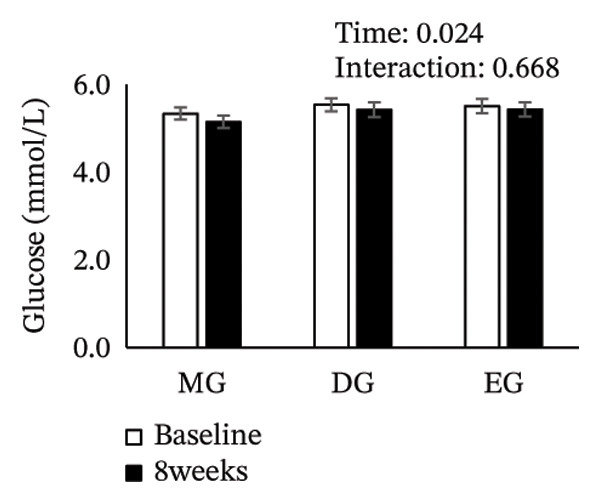
(a)

**Figure:**
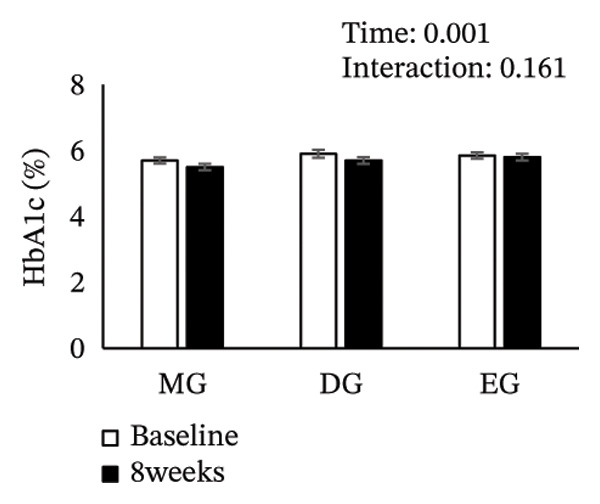
(b)

**Figure:**
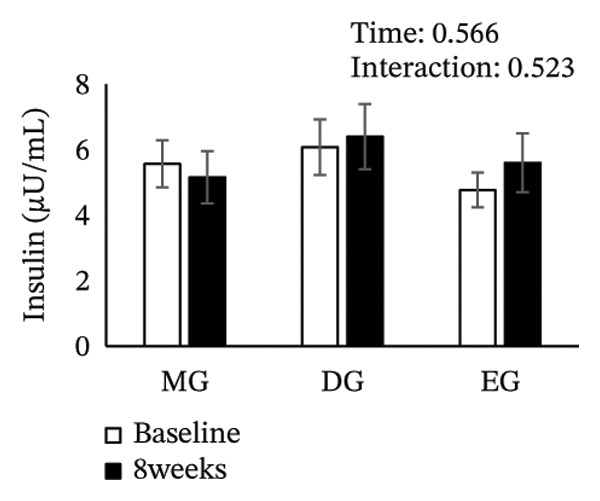
(c)

**Figure:**
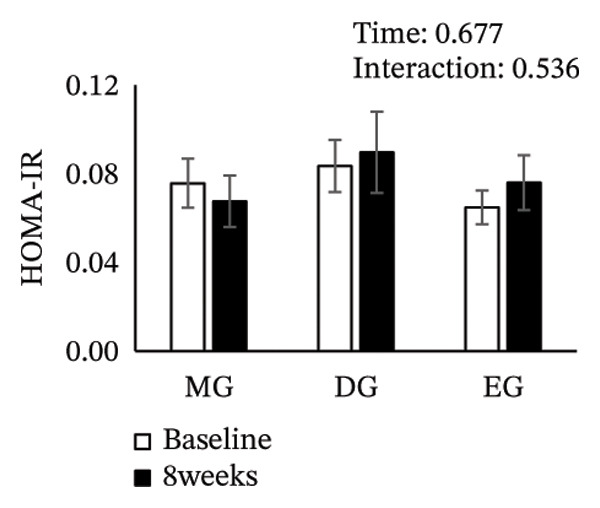
(d)

**Figure:**
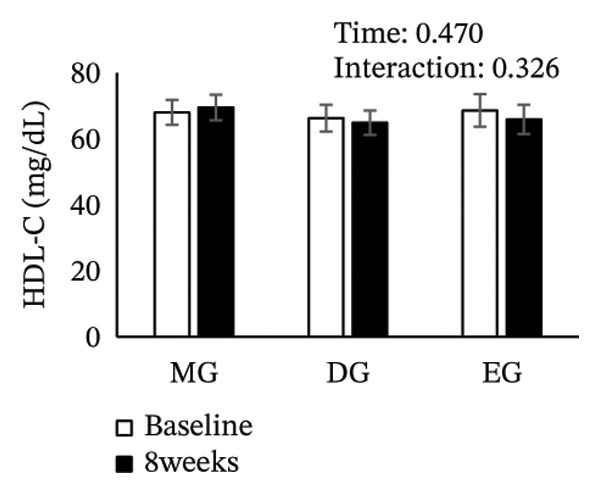
(e)

**Figure:**
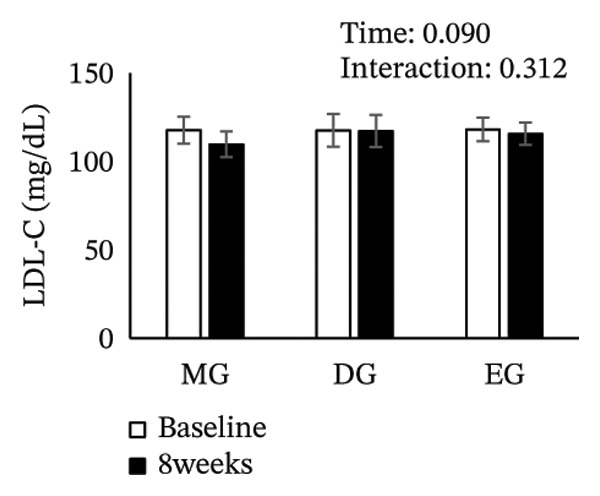
(f)

**Figure:**
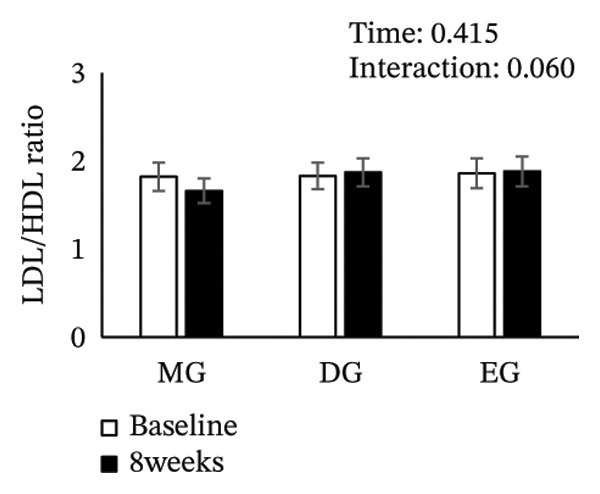
(g)

**Figure:**
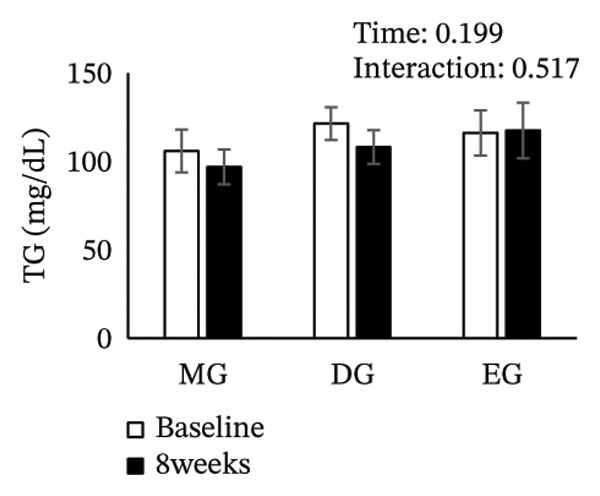
(h)

### 3.4. Effects of Green Tea–Intake Timing on Body Composition Parameters

Table 3 displays changes in BW, BMI, fat mass, and muscle mass. Two‐way ANOVA revealed no significant effects of group × time interactions for BW, BMI, fat mass, and muscle mass. However, significant main effects of time were detected for BW, fat mass, and muscle mass.

**TABLE 3:** Changes in body composition at baseline and after 8 weeks.

				**p** **value**	
		**Baseline**	**8 weeks**	**Time**	**Time** **×** **group**

BW (kg)	MG	58.6 ± 9.7	58.2 ± 9.2	0.030	0.751
	DG	63.5 ± 10.5	63.0 ± 10.3		
	EG	62.1 ± 9.9	61.9 ± 10.0		

BMI (kg/m^2^)	MG	22.1 ± 2.8	22.0 ± 2.6	0.056	0.888
	DG	23.5 ± 2.4	23.3 ± 2.4		
	EG	23.0 ± 2.4	22.9 ± 2.4		

Fat mass (kg)	MG	16.1 ± 5.0	15.2 ± 4.9	0.001	0.705
	DG	20.7 ± 4.4	19.6 ± 4.6		
	EG	17.4 ± 4.9	16.7 ± 4.8		

Muscle mass (kg)	MG	23.1 ± 4.8	23.4 ± 4.6	0.027	0.977
	DG	23.3 ± 5.6	23.6 ± 5.4		
	EG	24.4 ± 5.2	24.7 ± 5.3		

*Note:* Values are expressed as the mean ± standard error.

## 4. Discussion

To our knowledge, this is the first study to examine the chronic effects of green tea–intake timing on glucose and lipid metabolism in older adults. The principal finding was that regardless of the timing of green tea intake, the HbA1c level, blood glucose level, and fat mass were significantly reduced, while the muscle mass increased. These results indicate that continuous green tea consumption improves the glucose tolerance and body composition parameters regardless of intake timing.

Epigallocatechin gallate (EGCG), a major catechin in green tea, has been shown to improve glucose tolerance through several mechanisms. These mechanisms include suppressing hepatic gene expression of gluconeogenic enzymes (G6Pase and PEPCK), inhibiting the activity of carbohydrate‐digesting enzymes (α‐amylase and α‐glucosidase) to reduce glucose absorption, and promoting cellular glucose uptake via AMP‐activated protein kinase activation. Collectively, these mechanisms enhance glucose tolerance [17, 27, 28]. Previous studies have reported a reduction in fasting glucose and HbA1c levels with green tea consumption, and these findings are consistent with our results [24, 29]. A previous study has reported that when green tea is consumed acutely, its effect in reducing postprandial blood glucose levels is more effective at dinner than at breakfast [22]. Therefore, it was hypothesized that even with continuous green tea consumption, the EG would show better glucose tolerance than the MG and DG. However, the comparable outcomes obtained regardless of the timing of green tea consumption do not support our initial hypothesis. Furthermore, we had concerns about green tea consumption in the evening through to nighttime. Specifically, the caffeine in green tea may reduce sleep quality [30, 31], potentially adversely affecting glucose tolerance. Indeed, sleep deprivation and poor sleep quality have been reported to be associated with impaired glucose tolerance [32, 33]. Therefore, considering the diurnal variation in glucose tolerance, which declines from evening to night [11], while evening intake was expected to be most effective for improving glucose tolerance, it was also anticipated that this effect might be partially offset by sleep problems associated with caffeine intake. In this study, sleep quality before and after the intervention was evaluated using the Pittsburgh Sleep Quality Index. The results showed no significant differences in either the time × group interaction or the main effect of time. Moreover, there was no significant difference in sleep duration for each group before and after the intervention (Table S1). These results indicate that green tea consumption in EG did not negatively affect sleep quality. Green tea contains both caffeine and L‐theanine; the anxiety‐ and depression‐alleviating effects of theanine may have attenuated the effects of caffeine on sleep quality [34, 35]. We also evaluated the Geriatric Depression Scale‐15 and the World Health Organization‐Five Well‐Being Index as secondary outcome measures. However, no significant changes were observed in these measures following the intervention (Table S1). Therefore, the findings suggest that the effects of caffeine and theanine contained in green tea are unlikely to have influenced the results of the psychological assessment scale.

In this study, the HbA1c level decreased in all groups regardless of intake timing, but the underlying mechanisms may differ. The reductions observed in the MG and DG likely involve the second‐meal effect, that is, glucose tolerance improves after a subsequent meal due to the preceding meal. One mechanism involves enhanced β‐cell responsiveness during the second meal, induced by the earlier meal [36–40]. This effect is not limited to the period immediately following a meal and can persist for several hours [41]. Therefore, consuming green tea with glucose tolerance‐enhancing properties in the morning or day may improve postprandial control at lunch and dinner, contributing to HbA1c level reduction. On the contrary, the HbA1c level decrease in the EG appears to be related to diurnal variations in glucose tolerance. Glucose tolerance typically declines throughout the day, with higher postprandial glucose level at dinner than at breakfast [9]. Therefore, evening green tea consumption may improve glycemia during periods of reduced tolerance and consistent intake could result in long‐term improvement in postprandial hyperglycemia, lowering the HbA1c level [42]. This finding suggests that different mechanisms may have contributed to improved glucose tolerance at each intake time point, resulting in comparable outcomes regardless of the timing of green tea consumption.

EGCG improves lipid metabolism by interfering with cholesterol absorption through suppression of micellar solubilization of cholesterol and downregulation of genes involved in gluconeogenesis and lipid synthesis [43–46], as well as modulation of FoxO3a/HNF1*α* to inhibit PCSK9 activity, thereby enhancing hepatic LDL receptor‐mediated uptake and lowering the LDL‐C level [47]. Furthermore, EGCG may suppress the expression of sterol regulatory element‐binding protein‐1c, a transcription factor regulating lipid synthesis [48]. In fact, a meta‐analysis has shown that consuming 145–3000 mg of green tea catechins daily for 3–24 weeks lowers the LDL‐C level [49]. In the present study, we evaluated the effects of catechin intake at a similar range; however, no significant differences in LDL‐C levels were observed among the groups. This lack of effect may be attributed to the fact that baseline LDL‐C levels in our study population were within the normal range, potentially restricting the effect of catechins in improving lipid metabolism.

The reduction in BW and fat mass observed in this study is consistent with previous study findings [43–46, 50–53]. This result may be attributed to catechin‐mediated enhancement of fat oxidation, increased diet‐induced thermogenesis, and stimulation of brown adipose tissue activity [50, 51]. The simultaneous decrease in BW and increase in muscle mass represent a particularly intriguing finding. The observation suggests that the reduction in fat mass exceeded the increase in muscle mass, resulting in an overall decrease in BW. The observed increases in muscle mass may be attributed to several catechin‐mediated mechanisms. Tea catechins have been shown to reduce oxidative stress and maintain mitochondrial function in skeletal muscle [54], while promoting myogenesis by inducing the expression of myogenin and muscle creatine kinase [55]. Additionally, epicatechin has been shown to reduce myostatin and β‐galactosidase levels while elevating the levels of markers associated with muscle growth [56]. Indeed, 12‐week supplementation of tannase‐treated green tea extract rich in epicatechin and gallic acid significantly increased muscle strength, grip strength, and muscle mass in individuals aged ≥ 60 years, independent of exercise performance [57]. These results suggest that the muscle mass gains observed in the present study may have resulted from catechin‐induced antioxidant activity, myostatin suppression, and enhanced myogenesis.

Potential confounding factors include physical activity and diet [58, 59]. To minimize their effects, the participants were instructed to maintain stable lifestyle habits throughout the study. No significant differences were observed in major nutrient intake (energy, protein, fat, and carbohydrate), evaluated using the FFQ, before and after intervention across the groups. Additionally, no significant differences were found in total physical activity after intervention, as measured using the International Physical Activity Questionnaire, compared with baseline values in each group. These findings strengthen the inference that improvements in lipid and glucose metabolism are attributable to green tea consumption rather than lifestyle factors.

Our findings may hold clinical relevance. In patients with diabetes, a 0.9% reduction in HbA1c level reduces nephropathy and retinopathy risk by 20% and 13%, respectively [60]. Although this study included participants without diagnosed diabetes, kidney disease, or dyslipidemia, making it difficult to assess clinical significance within the normal range, the observed improvements may contribute to prevention of NCDs in those at high risk. A notable strength of this study is the use of a commercially available beverage, supporting its intake as a feasible preventive strategy. Additionally, adherence was high (≥ 90% in all groups), enhancing the reliability of the findings. However, this study has certain limitations. First, participants were restricted to healthy individuals without diabetes, leaving the effects of long‐term green tea consumption timing uncertain for populations with NCDs or across different age groups. Future interventional studies involving more diverse participants are necessary to enhance the generalizability and validity of these findings. Second, designing a placebo‐controlled group was challenging. As many participants habitually consumed green tea, establishing a nonconsumption group and restricting intake over the long term were not feasible. Previous studies have demonstrated the health benefits of green tea; therefore, the primary objective of this study was not to verify the effects of green tea per se but rather to clarify the differential effects of the timing of consumption [21, 61, 62]. Therefore, we adopted a pre–post intervention design. We acknowledge that without a placebo control group, we must be cautious about attributing the observed changes solely to the main effects of green tea. Future studies should recruit larger participant cohorts and adopt designs incorporating placebo‐controlled groups to more rigorously evaluate the effects of green tea. Third, the FFQ was used to evaluate energy and nutrient consumption, which may have limitations in precisely capturing the amount of food consumed per meal [63]. Consequently, the observed results could be attributed to decreased dietary and caloric consumption beyond the test beverages, changes that may not have been adequately captured by the FFQ.

## 5. Conclusion

In older adults, green tea consumption over 8 weeks reduced the glucose level, HbA1c level, and fat mass, while increasing muscle mass regardless of intake timing. Our results indicate that individuals can expect similar improvements in glucose tolerance and body composition parameters from green tea consumption regardless of intake timing.

## Author Contributions

Saeka Fuke contributed to study conceptualization, investigation, data analysis, and draft writing. Masaki Takahashi contributed to study conceptualization, data analysis, investigation, funding acquisition, and manuscript writing–review and editing. Kyoko Fujihira contributed to study conceptualization, investigation, and manuscript writing–review and editing.

## Funding

This work was supported by a research grant from the Hachiro Honjo Ocha Foundation (approval number: G24‐0025).

## Ethics Statement

This study was conducted in accordance with the tenets of the Declaration of Helsinki and approved by the ethics committee of the Institute of Science Tokyo (Tokyo, Japan) (approval number: 2024213).

## Conflicts of Interest

Masaki Takahashi received a research grant from the Hachiro Honjo Ocha Foundation. SF and KF declare no conflicts of interest.

## Supporting Information

CONSORT checklist for randomized controlled trials.

Table S1. Changes in PSQI, sleep duration, WHO5, and GDS10 at baseline and after 8 weeks. PSQI; Pittsburgh Sleep Quality Index; GDS‐15, Geriatric Depression Scale‐15; WHO‐5, World Health Organization‐Five Well‐Being Index.

## Supporting information


**Supporting Information** Additional supporting information can be found online in the Supporting Information section.

## Data Availability

The data that support the findings of this study are available from the corresponding author upon reasonable request.
