# Supplementary material for: Effects of Green Tea–Intake Timing on Glucose and Lipid Metabolism in Older Adults: An 8‐Week Randomized Controlled Trial
Source: J Nutr Metab. 2026 Apr 7;2026:2301278. doi: 10.1155/jnme/2301278 (PMC13054514; doi:10.1155/jnme/2301278)
Supplement: Supplementary file 1 — Supporting Information Additional supporting information can be found online in the Supporting Information section. [file JNME-2026-2301278-s001.zip › Table S1.docx]

| **Table S1.** Changes in PSQI, sleep duration, WHO5 and GDS10 at baseline and after 8 weeks | | | | | | | |
| --- | --- | --- | --- | --- | --- | --- | --- |
|  |  | **Baseline** | | | **8 weeks** | | |
|  |  |  |  |  |  |  |  |
| PSQI (score) | MG | 4.5 | ± | 0.5 | 3.9 | ± | 0.6 |
|  | DG | 5.0 | ± | 0.5 | 5.5 | ± | 0.7 |
|  | EG | 5.0 | ± | 0.5 | 4.9 | ± | 0.5 |
| Sleep duration (h) | MG | 6.7 | ± | 0.3 | 6.8 | ± | 0.3 |
|  | DG | 7.0 | ± | 0.2 | 6.6 | ± | 0.3 |
|  | EG | 6.2 | ± | 0.3 | 6.2 | ± | 0.3 |
| WHO5 (score) | MG | 16.1 | ± | 1.1 | 16.1 | ± | 0.9 |
|  | DG | 16.2 | ± | 1.0 | 15.7 | ± | 1.3 |
|  | EG | 16.6 | ± | 0.9 | 18.0 | ± | 1.0 |
| GDS15 (score) | MG | 2.7 | ± | 0.8 | 2.7 | ± | 0.8 |
|  | DG | 3.1 | ± | 0.8 | 3.0 | ± | 0.7 |
|  | EG | 2.0 | ± | 1.0 | 1.8 | ± | 0.8 |

Values are expressed as mean ± standard error. Two-way ANOVAs revealed no significant effects of group × time interactions for PSQI, WHO5. Data that were non-normally distributed were analyzed using Wilcoxon’s t-test to examine differences within groups at baseline and 8 weeks. There was no significant difference in Sleep duration and GDS15 for each group before and after the intervention (Wilcoxon’s t-test). PSQI; Pittsburgh Sleep Quality Index; GDS-15, Geriatric Depression Scale-15; WHO-5, World Health Organization-Five Well-Being Index
